# Transcriptome Analysis of Potato (*Solanum tuberosum* L.) Seedlings with Varying Resistance Levels Reveals Diverse Molecular Pathways in Early Blight Resistance

**DOI:** 10.3390/plants14152422

**Published:** 2025-08-05

**Authors:** Jiangtao Li, Jie Li, Hongfei Shen, Rehemutula Gulimila, Yinghong Jiang, Hui Sun, Yan Wu, Binde Xing, Ruwei Yang, Yi Liu

**Affiliations:** 1 Urumqi Comprehensive Experimental Station, Xinjiang Academy of Agricultural Science, Urumqi 830013, China; xjnkyljt@163.com (J.L.); hongfeiscience@163.com (H.S.); qiaokeli813@163.com (R.G.); forjyh@126.com (Y.J.); nkysh189@163.com (H.S.); 15099502765@163.com (Y.W.); xingbd91@163.com (B.X.); 2 Xinjiang Agricultural Vocational and Technical University, Changji 831100, China; xjnyzyjsdxlj@163.com

**Keywords:** virus-free potato, early blight, biotic stress, transcriptional response, WGCNA, hub genes, differentially expressed genes

## Abstract

Early blight, caused by the pathogen *Alternaria solani*, is a major fungal disease impacting potato production globally, with reported yield losses of up to 40% in susceptible varieties. As one of the most common diseases affecting potatoes, its incidence has been steadily increasing year after year. This study aimed to elucidate the molecular mechanisms underlying resistance to early blight by comparing gene expression profiles in resistant (B1) and susceptible (D30) potato seedlings. Transcriptome sequencing was conducted at three time points post-infection (3, 7, and 10 dpi) to identify differentially expressed genes (DEGs). Weighted Gene Co-expression Network Analysis (WGCNA) and pathway enrichment analyses were performed to explore resistance-associated pathways and hub genes. Over 11,537 DEGs were identified, with the highest number observed at 10 dpi. Genes such as *LOC102603761* and *LOC102573998* were significantly differentially expressed across multiple comparisons. In the resistant B1 variety, upregulated genes were enriched in plant–pathogen interaction, MAPK signaling, hormonal signaling, and secondary metabolite biosynthesis pathways, particularly flavonoid biosynthesis, which likely contributes to biochemical defense against *A. solani*. WGCNA identified 24 distinct modules, with hub transcription factors (e.g., *WRKY33*, *MYB*, and *NAC*) as key regulators of resistance. These findings highlight critical molecular pathways and candidate genes involved in early blight resistance, providing a foundation for further functional studies and breeding strategies to enhance potato resilience.

## 1. Introduction

Potato is a versatile crop for food, feed, and vegetable purposes. It is the fourth largest food crop in the world and an industrial raw material [1,2]. Potato planting area in China has consistently increased over a period of time, reaching 4.76 million hectares per year with a total yield of 90 million tons, accounting for 24% and 23% of the global cultivation area and total production, respectively, making it the world leader in both potato cultivation and production [3,4]. As one of the important crops for the adjustment of crop planting structure in many potato growing regions and to increase farmer income, potato cultivation has a huge scope for research and development. However, potatoes are vulnerable to diseases such as late blight (*Phytophthora infestans*), early blight (*Alternaria solani*), and bacterial wilt (*Ralstonia solanacearum*), which can significantly reduce tuber yield, quality, and storability [5,6]. Some researchers have reported annual yield declines of up to 30% due to various biotic stresses, including insects, bacteria, or fungal diseases [7,8].

Early blight is a polycyclic disease caused by *A. solani* and which survives in the form of spores and mycelium in infected plants [9,10]. This pathogen can complete several life cycles of infection in a single season, causing great risk to other plants on the farm [11]. Early blight fungi belong to the *Alternaria* genus. *A. solani* is a necrotrophic fungus mainly responsible for the yellowing of leaves, followed by large-scale necrosis in infected plants and subsequent defoliation [12]. Symptomatic lesions on leaves of early blight infection are characterized by dark concentric-ring lesions that are restricted within leaf veins [11]. At the cellular level, *A. solani* infection triggers cell wall degradation through the secretion of cell wall-degrading enzymes, oxidative stress via reactive oxygen species (ROS) accumulation, and programmed cell death (apoptosis-like processes) in potato leaf tissues, contributing to tissue necrosis and reduced photosynthetic capacity [13,14].

In contrast, late blight presents as water-soaked lesions that rapidly spread, causing extensive foliar and tuber damage, potentially resulting in complete crop failure under conducive conditions. While early blight thrives in warm, dry conditions, late blight favors cool, wet environments, necessitating distinct management strategies. This study focuses on early blight resistance, addressing the urgent need to identify genetic mechanisms to mitigate its impact on potato production [15,16]. To counteract these cellular changes, plants activate defense responses, including the expression of pathogenesis-related (PR) genes, antioxidant enzymes, and secondary metabolites like phenolic compounds, which mitigate oxidative damage and reinforce cell walls.

The disease can infect plants in a variety of climatic conditions and can spread rapidly if environmental conditions are favorable [17]. Early blight can mainly harm the leaves, stems, and tubers, reducing yields and causing tuber rot during storage [18,19,20]. Recent studies highlight that *A. solani* infection triggers extensive transcriptomic reprogramming in potato, including the upregulation of defense-related genes such as pathogenesis-related (PR) genes and those involved in phenylpropanoid biosynthesis, which contribute to cell wall reinforcement and resistance to necrosis [21]. Additionally, *A. solani* induces oxidative stress through reactive oxygen species (ROS) accumulation, disrupting cellular homeostasis. In a recent study, multi-omics was used to identify a specific effector, *AsCEP20*, in *A. solani*, and further experiments showed that *AsCEP20* suppresses *StFtsH4*-mediated potato disease resistance [22]. These molecular responses underscore the importance of identifying differentially expressed genes (DEGs) to develop resistant potato cultivars, as explored in this study through transcriptomic analysis to elucidate genetic mechanisms for early blight resistance. During cultivation, potato production is affected by numerous biotic and abiotic factors, resulting in significant losses for potato farmers worldwide [23,24,25].

Various experiments have been carried out to control pathogenic activities on potatoes. Most of the studies are based on the isolation and identification of pathogenic microbes. Recently, researchers have studied the impact of various fungicide applications. Breeding and cultivation of resistant varieties, as compared to cultural practices and application of fungicides, is the most efficient, economic, and environmentally friendly approach for early blight control. Additionally, various fungicides (copper and sulfur-based) and biocontrol agents (*Bacillus subtilis*, *Trichoderma* spp., and *Pseudomonas fluorescens*) have been utilized, all aimed at preventing and managing this disease [26,27,28]. In recent studies, some organic methods, including plant extracts, botanicals, and beneficial microbes, were prioritized to control disease due to environmental concerns [29]. For example, Murmu et al. (2015) applied fresh garlic (*Allium sativum*) extract that significantly reduced the severity of potato early blight [30]. Kumar et al. [31] used *Trichoderma viride* and fungicides to manage potato early blight. The findings showed that the application of these organic controls improved the overall quality of the tubers [31]. In a previous study, *Bacillus subtilis* was reported to have antagonistic effects against several varieties of pathogenic fungi [20].

Recent studies on the molecular level indicated that the breeding of resistant varieties is one of the important measures to control early blight in potatoes. Xue et al. (2019) conducted a field experiment with 217 tetraploid potato varieties to evaluate their resistance to early blight and found that most resistant and medium-resistant varieties matured late, while the most susceptible varieties matured early, with a few exceptions [32]. Sajeevan et al. [33] analyzed the transcriptome profiling of three different potato cultivars, ‘Magnum Bonum’, ‘Kuras’, and ‘Desiree’, infected with the potato blight pathogen. They found that transcription factors such as *C_2_H_2_*, *MYB*, *WRKY*, *ERF*, and *bHLH* showed an upregulated trend after infection. In addition, some genes from jasmonic acid (JA) and ethylene biosynthesis pathways were highly upregulated. The Mevalonate (MVA) pathway and terpene biosynthesis genes were upregulated in different potato varieties at various time points [33]. Brouwer et al. [34] analyzed the transcriptomic regulation at different time points after *A. solani* inoculation in leaves of the susceptible cultivar ‘Desiree’. The outcomes revealed that salicylic acid (SA) was involved in regulating the development of symptoms in potato leaves and tubers [34]. SA signaling is necessary for defense against the early blight pathogen. However, there are a few comparative studies where highly contrasting plant varieties were used to map the transcript regulation and find the genetic network involved in resistance. Some recent studies revealed that there are some cultivars that have different levels of early blight resistance. The resistance in potatoes is suggested to be quantitative due to the lack of specific resistance (R) genes that regulate infection [12,35].

Histopathological studies revealed that leaflets of Aracy (resistant), Delta (moderately resistant), and Bintje (susceptible) cultivars have varying levels of early blight resistance [36]. The ultrastructural observation showed that penetration in the susceptible variety occurred most frequently through the junctions of the epidermal cells. Current control methods, such as fungicide applications, are costly, environmentally unsustainable, and often ineffective against evolving pathogen strains. Consequently, developing resistant potato cultivars through genetic approaches is a critical strategy to mitigate these losses. Comparative transcriptomic profiling of resistant, moderately resistant, and susceptible cultivars offers a powerful approach to identify differentially expressed genes (DEGs) and pathways, such as those involved in cell wall reinforcement and defense signaling, that underpin resistance mechanisms. Hence, it is of great interest to identify such varieties and use them for comparative profiling to identify candidate genes and pathways contributing to resistance in potato varieties. In this study, we performed a transcriptomic analysis of resistant (B1) and susceptible (D30) potato varieties to investigate the defense responses to *A. solani* using in-depth RNA sequencing (RNA-seq), and their responses to infection were measured at three different time points. This study aims to address this challenge by conducting phenotypic screening to identify potato varieties with diverse levels of early blight resistance, followed by in-depth RNA sequencing (RNA-seq) at multiple time points to capture the dynamic regulation of genes and molecular pathways. Bioinformatic analyses, including Gene Ontology (GO), Kyoto Encyclopedia of Genes and Genomes (KEGG), Weighted Gene Co-expression Network Analysis (WGCNA), and pathway analysis, were performed to predict diverse genetic regulatory mechanisms underlying resistance. Comparative transcriptomic profiling of resistant and susceptible cultivars enables the identification of DEGs and pathways, paving the way for marker-assisted breeding to enhance early blight resistance in potato.

## 2. Results

### 2.1. Resistance of Virus-Free Potato Seedlings to Early Blight

**Figure 1:**
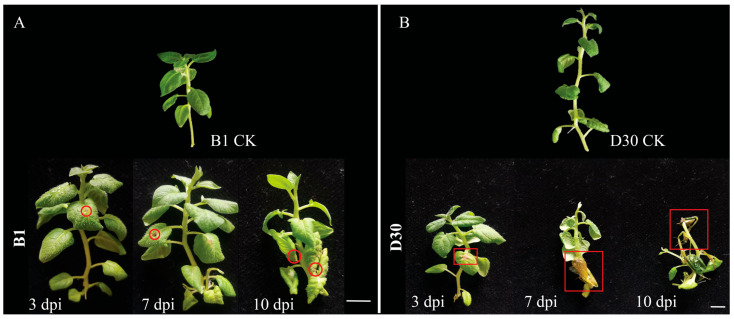
Phenotypic difference between ‘B1’ and ‘D30’ potato (*S. tuberosum*) varieties. The disease symptoms in both varieties are highlighted with a red circle or box. (**A**): resistant variety; (B1) and (**B**): response of sensitive variety (D30).

Phenotypic observation was made at 3, 7, and 10 dpi to investigate the infection of early blight disease and potential resistance mechanisms in sensitive and resistant virus-free seedlings of potato. It was observed that, under the same cultivation conditions, the growth of virus-free ‘B1’ seedlings was better than ‘D30’. After infection by *A. solani*, ‘B1’ showed strong resistance, and there was little difference between both varieties at the early stage (3 dpi) of infection. The lower leaves of the virus-free seedlings showed a slight yellow trend after 7 dpi. After 3 days of infection by *A. solani*, the leaves of ‘D30’ showed a yellow leaf phenomenon, and with the passage of time, after 7 days of treatment, the leaves of the lower part of the seedling curled and yellowed. By the 10th day, the upper part of the seedling not only showed yellowing and leaf curling, but even the stems showed yellowing. As can be seen from Figure 1, B1 showed resistance to *A. solani*, while D30 showed weak resistance to *A. solani*, belonging to the sensitive potato.

### 2.2. Details of Transcriptome Sequencing Data

Control (B1-CK and D30-CK) and infected potato leaves were collected at 3 (B1/D30-3d) days, 7 (B1/D30-7d) days, and 10 (B1/D30-10d) days post-infection for transcriptome sequencing. Three biological repeats were maintained. A total of eight treatment groups with multiple RNA-seq libraries were constructed, and the standard protocol was followed for downstream analysis. The total raw reads obtained varied from 39,845,276 to 51,370,438 among all the treatment groups. After filtration, the number of clean reads varied from 38,393,288 to 49,613,630, which accounted for more than 96% of these high-quality sequences. The mapping rate of each sample ranged from 85.92% to 88.87%. The overall data presented in Table S2 showed that the sequence characteristics were good enough to meet the requirements of subsequent data assembly and processing. Whole-genome protein sequences from existing reference genomes were obtained and compared with databases such as KEGG (https://www.genome.jp/kegg/) (accessed on 15 July 2024) [37], NR, SwissProt (https://www.expasy.org/resources/uniprotkb-swiss-prot) (accessed on 19 July 2024), KOG (http://genome.jgi-psf.org/help/kogbrowser.jsf) (accessed on 3 August 2024), GO, etc., to predict the function of the proteins. The details of the number of genes mapped on the database are shown in Table S3.

### 2.3. Differential Expression Gene Analysis

**Figure 2:**
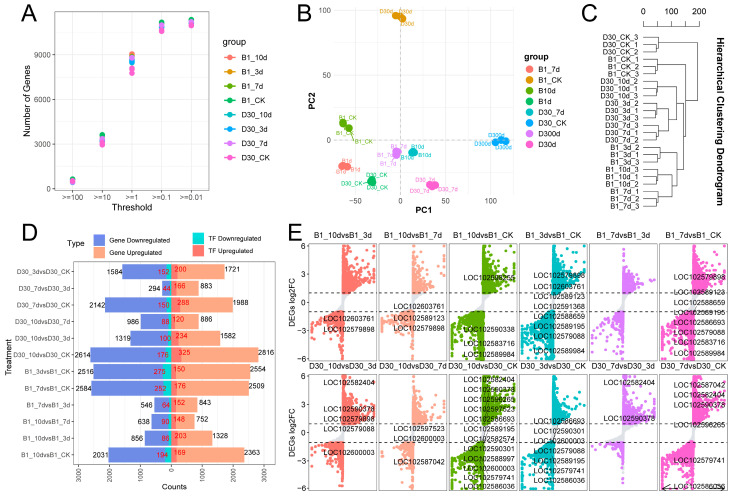
Overview of differentially expressed gene results: (**A**) Number of genes on threshold level among treatment groups. Figure illustrates the magnitude of transcriptional changes induced by each treatment, highlighting the number of upregulated and downregulated genes in pairwise comparisons, thereby providing insight into the treatment-specific impacts on gene expression. (**B**) Principal component analysis (PCA) and sample cluster analysis. The first two principal components (PC1 and PC2) capture the major sources of variation in the dataset, allowing visualization of sample similarity and separation between treatment groups. (**C**) Clustering dendrogram of all treatment groups. The dendrogram reflects the overall similarity in gene expression patterns among samples, with closely related clusters indicating shared transcriptional responses. (**D**) Differentially expressed genes and transcription factors in different comparison groups. (**E**) Volcano plot for each group to show the distribution of genes.

The number of genes expressed across different thresholds in the two potato varieties (B1 and D30) under control (CK) and post-inoculation conditions at 3, 7, and 10 days (dpi) is shown in Figure 2A. To explore the dynamic changes of responsive genes after inoculation, we analyzed the number of expressed genes under different FPKM thresholds across various time points in both control and inoculated groups of the two potato varieties. As the FPKM expression threshold decreased, the number of detected genes in each treatment group increased significantly. For each variety, the number of expressed genes in the control and inoculated groups showed certain differences over time, suggesting that inoculation treatment may affect the number of expressed genes, and the duration of inoculation may influence their expression patterns. Under the same threshold setting, differences in the number of genes between B1 and D30 at the same time point indicate that the two varieties exhibit distinct gene expression patterns during the tolerance period.

Based on the results associated with differentially expressed genes (DEGs), principal component analysis (PCA) revealed the dynamics of gene expression in *A. solani*-infected seedlings of ‘B1’ and ‘D30’ potato varieties. The control groups in PCA were clustered separately, reflecting intrinsic differences in their baseline gene expression profiles and responses to infection. The separation within both varieties at different time points post-inoculation reveals that gene expression response evolves dynamically after inoculation (Figure 2B). The distinct groups in the Hierarchical Clustering Dendrogram (Figure 2C) show the genetic differences between the two varieties at the transcript levels. The clear cluster separation by time points (3d, 7d, 10d) was observed in both varieties. Overall, this different analysis showed the gene expression response post-inoculation in varieties.

The number of differentially expressed genes (DEGs) and transcription factors (TFs) under various comparisons is shown in Figure 2D. The detailed analysis of DEGs showed that the highest number of DEGs was observed at 10dpi. For instance, D30_10d vs. D30_CK: 2614 genes upregulated and 275 downregulated. In the early stage of infection, fewer DEGs were observed. ‘D30’ consistently showed more DEGs compared to ‘B1’, especially at 7d and 10d dpi. The observations showed that transcription factors (TFs) are also differentially regulated, with more TFs upregulated in ‘D30’. Volcano plots comparing the log2 fold-change (log2FC) of DEGs across various pairwise comparisons were generated to gain more insights into DEGs (Figure 2E). The results in volcano plots showed that specific genes, such as *LOC102603761*, *LOC102573998*, and others, are prominently differentially expressed in multiple comparisons, indicating their significant involvement in the response to *A. solani* infection in both varieties. These genes consistently show strong upregulation. The compression in both varieties highlighted that ‘D30’ consistently showed a broader distribution of log2FC values compared to ‘B1’, particularly at later time points (7d and 10d dpi). Interestingly, D30 exhibits a higher density of highly upregulated genes compared to ‘B1’, indicating the role of multiple genes in disease response.

### 2.4. Functional Enrichment Analysis of Differentially Expressed Genes GO

**Figure 3:**
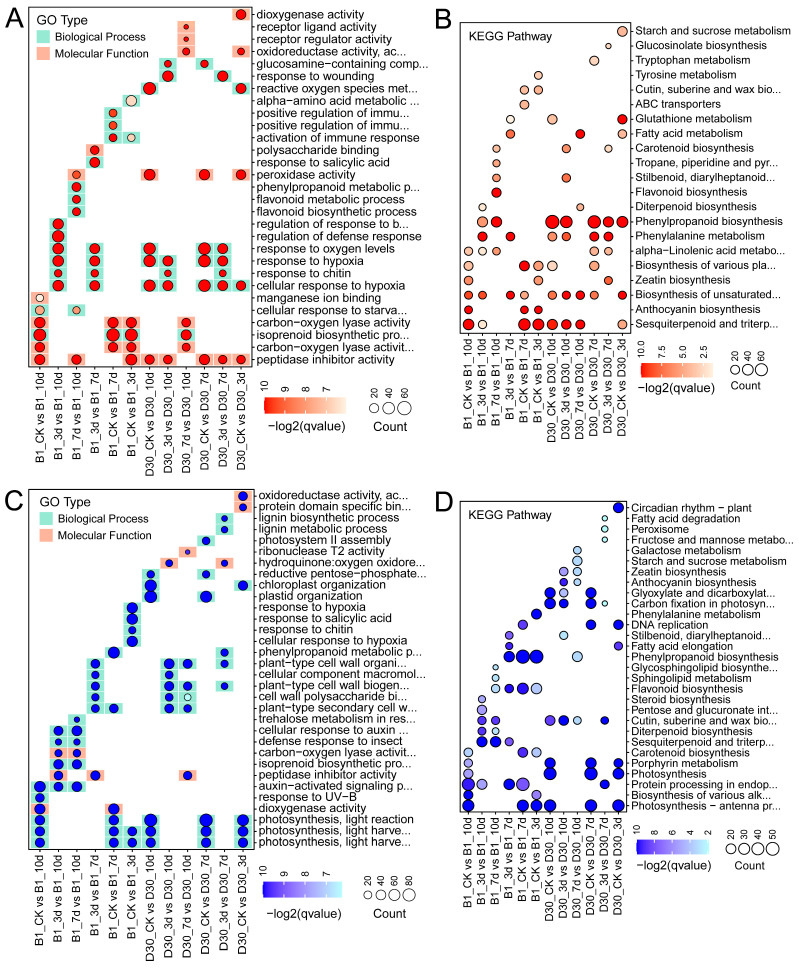
GO and KEGG analysis for DEGs identified in different treatment groups. Enrichment analysis of differentially expressed genes (DEGs) in resistant (B1) and sensitive (D30) varieties of potato (*S. tuberosum*) infected with *A. solani.* (**A**,**B**) show GO and KEGG enrichment for upregulated DEGs, respectively, while (**C**,**D**) show GO and KEGG enrichment for downregulated DEGs.

Results from enrichment analyses of differentially expressed genes (DEGs) for GO (Gene Ontology) and KEGG (Kyoto Encyclopedia of Genes and Genomes) pathways are presented in Figure 3. In the resistant variety (B1), upregulated DEGs were enriched in stress-related biological processes, such as response to oxidative stress, cell wall modification, and phenylpropanoid biosynthesis, indicating a strong defense response (Figure 3A). The KEGG analysis (Figure 3B) reveals pathways significantly enriched among upregulated DEGs. In B1, key defense pathways, such as phenylpropanoid biosynthesis and plant–pathogen interaction, are prominent, particularly at 7 and 10 dpi, signifying the defense mechanisms against *A. solani*. In contrast, D30 shows limited enrichment in these pathways, underscoring its susceptibility. The resistant variety demonstrated a more targeted and sustained activation of metabolic and defense pathways compared to the sensitive variety. In addition, the GO and KEGG analyses of downregulated genes were also performed (Figure 3C,D). The GO analysis of downregulated DEGs showed that, in ‘B1’, there is less downregulation of critical defense-related genes. However, in D30, processes related to photosynthesis and metabolic regulation are significantly downregulated. The KEGG analysis (Figure 3D) for downregulated DEGs highlights disrupted pathways in ‘D30’, such as photosynthesis and carbon metabolism, which may contribute to its susceptibility. In contrast, B1 maintains these pathways to a greater extent, supporting its resistance. The enrichment analysis underscores the differential responses of the resistant (B1) and sensitive (D30) varieties to *A. solani*. ‘B1’ demonstrates a strong activation of defense-related pathways, whereas ‘D30’ shows significant downregulation of key physiological pathways.

### 2.5. Differential Gene Expression Analysis and Trend Cluster Analysis

**Figure 4:**
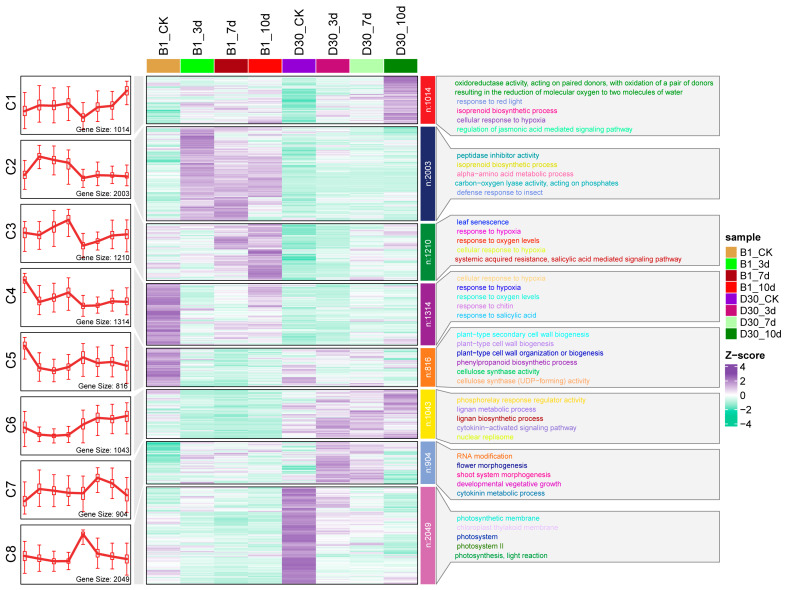
Trend cluster analysis of DEGs divided into 8 clusters (C1–C8) in B1-resistant and D30-sensitive lines across control and 3, 7, and 10 days post-inoculation. The gene number varies in each cluster. The C1 (1014), C2 (2003), C3 (1210), C4 (1314), C4 (1314), C5 (816), C6 (1043), C7 (904), and C8 (2049).

Trend cluster analysis was performed on all DEGs in both the resistant (B1) and sensitive (D30) varieties under various conditions, including control (CK) and at 3, 7, and 10-days post-inoculation, which were divided into eight clusters (C1 to C8), reflecting different biological processes in response to inoculation (Figure 4). The Z-scores of gene expression in the heatmap show downregulation (purple) and upregulation (green) across the experimental timeline (Figure 4). Cluster C1, comprising 1014 genes, shows a consistent decrease in expression over time in both lines. Cluster C2 is particularly important, as it is strongly induced in the ‘B1’-resistant varieties but not in the D30-sensitive variety. C2 cluster contains 2003 genes, which were primarily enriched in peptidase inhibitor activity and biosynthesis processes of metabolites. Peptidase inhibitors are known to play a crucial role in defense by inhibiting proteases that pathogens use to invade plant tissues. Biosynthesis of other compounds contributes to the production of isoprenoids, which are essential for signaling and defense responses (Figure 4). The selective activation of these genes in the ‘B1’ line suggests that they play a key role in its resistance mechanism. Cluster C3, with 1210 genes, exhibits significant induction at early to mid-time points (3d and 7d). These genes are enriched in processes such as leaf senescence and hypoxia response, indicating an adaptive response to oxygen deprivation, which is often associated with biotic stress (Figure 4).

Cluster C4, containing 1314 genes, shows moderate induction in the ‘B1’-resistant variety but weaker activation in the ‘D30’-sensitive variety. These genes are enriched in responses to chitin, a key component of fungal pathogens, and the salicylic acid signaling pathway, which is critical for systemic acquired resistance (Figure 4). Cluster C5, with 816 genes, peaks in expression at the 10dpi, particularly in the ‘B1’ line. These genes are involved in plant cell wall biosynthesis and the phenylpropanoid pathway processes. Cluster C6, comprising 1043 genes, shows a gradual induction over time in both lines. Cluster C7, with 994 genes, has a sharp induction at the 7dpi and is enriched in RNA modification and flower morphogenesis, indicating active transcriptional regulation and potential stress-related developmental reprogramming (Figure 4). Finally, Cluster C8, the largest cluster with 2049 genes, shows strong late-stage induction, especially in the ‘B1’ line. These genes are enriched in photosynthesis-related processes, including the light reaction, suggesting recovery or maintenance of photosynthetic activity under stress conditions.

### 2.6. Regulatory Network Between Genes and TFs Based on WGCNA Analysis

**Figure 5:**
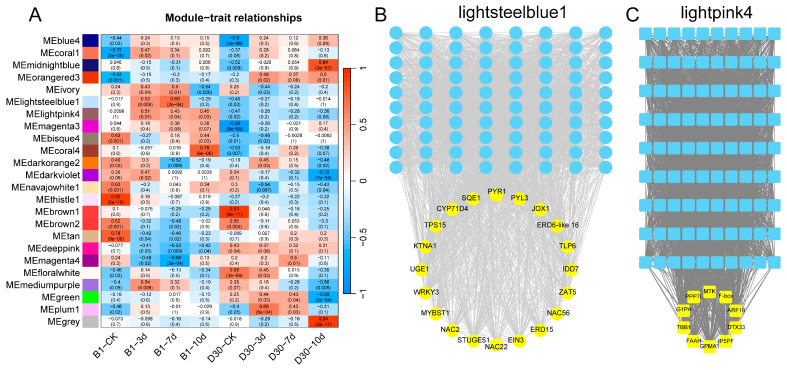
Regulatory network between genes and transcription factors (TFs) based on WGCNA analysis: (**A**) Module-trait relationships showing the correlation between gene modules and different time points. (**B**) Gene regulatory network of the lightsteelblue1 module, highlighting key transcription factors (yellow nodes) associated with disease resistance in the ‘B1’ variety. (**C**) Gene regulatory network of the lightpink4 module, showing key transcription factors (yellow nodes) linked to disease susceptibility in the ‘D30’.

To elucidate regulatory pathways and identify potential key genes involved in the pathogenesis and immune response of potato against *A. solani* infection, the identified genes were classified into 24 distinct modules through WGCNA. Two modules, lightsteelblue1 and lightpink4, were selected based on *p*-values for further co-expression network of hub gene analysis. The heatmap was generated to illustrate the correlation between different gene modules and potato varieties (‘B1’ and ‘D30’) at different time points (CK, 3, 7, and 10 dpi). Modules such as lightsteelblue1 and lightpink4 exhibit significant correlations with specific time points and varieties (Figure 5A). For example, the lightsteelblue1 module shows strong positive correlations with resistant variety ‘B1’ at specific time points, while lightpink4 shows distinct patterns linked to susceptibility traits in ‘D30’. The gene regulatory network from the lightsteelblue1 module is presented in Figure 5B. Blue nodes represent hub genes, while yellow nodes highlight transcription factors (TFs) identified as key regulators. TFs like *WRKY33*, *MYB*, and *NAC* are the key regulators. These genes and TFs are probably contributing to the disease resistance observed in the ‘B1’ variety. Regulatory network for the lightpink4 module is shown in Figure 5C. The results showed that TFs such as *AP2*, *ERF*, and *FAM* are the prominent regulators. The network suggests that these TFs might be associated with the disease susceptibility traits in the ‘D30’ variety.

### 2.7. Key Biosynthesis Pathway Associated with Differential Response to A. solani Infection

**Figure 6:**
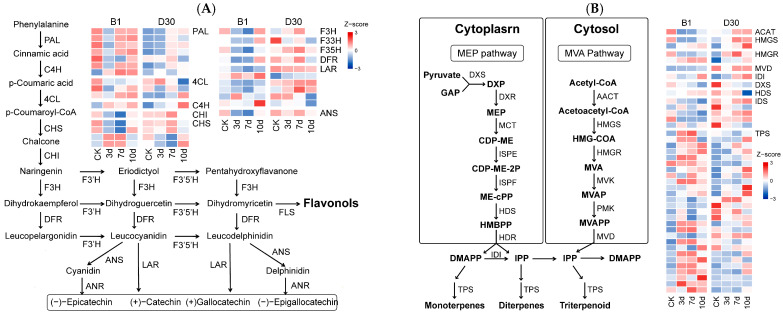
Expression profiles of related genes involved in different pathways: (**A**) DEGs in phenylpropanoid pathways. (**B**) MEP pathway in the cytoplasm and the MVA pathway genes and expression analysis at different times post-inoculation.

Differential expressions of pathway genes (Table S4) in disease-resistant (B1) and disease-susceptible (D30) potato varieties under *A. solani* infection are presented through a heatmap (Figure 6). Differential expressions of genes involved in phenylpropanoid and flavonoid biosynthesis pathways in ‘B1’ and ‘D30’ potato varieties at different time points (CK, 3, 7, and 10 dpi) presented critical information about the roles of these genes. The heatmaps display Z-scores representing gene expression levels (Figure 6). In the resistant variety ‘B1’, early activation of important pathway genes such as PAL (Phenylalanine ammonia-lyase), 4CL (4-coumarate-CoA ligase), and CHI (Chalcone isomerase) was observed, indicating a strong early metabolic response to pathogen infection. Genes downstream in the pathway, such as F3H (Flavanone 3-hydroxylase), F3′H (Flavonoid 3′-hydroxylase), DFR (Dihydroflavonol-4-reductase), and ANS (Anthocyanidin synthase), showed higher expression in ‘B1’, leading to increased production of flavonoid compounds like catechins and anthocyanins, which are associated with pathogen defense. In contrast, the susceptible variety ‘D30’ displayed delayed and lower expression levels of these key genes, particularly 4CL (*LOC102581073*, *LOC102589212*, and *LOC102594991*), DFR (*LOC102587107*), and ANS (*LOC102598323*), suggesting a lower ability to produce defense-related secondary metabolites. These results suggest that the resistant variety ‘B1’ leverages the phenylpropanoid and flavonoid pathways more effectively to mount a biochemical defense against *A. solani* infection.

The MEP (Methylerythritol Phosphate) pathway in the cytoplasm and the MVA (Mevalonate) pathway in the cytosol are critical pathways in plants. The DEGs in these pathways are shown in Figure 6B. In the resistant variety ‘B1’, early time points (3- and 7-dpi) showed significant upregulation of critical genes in the MEP pathway, such as DXS (1-deoxy-D-xylulose-5-phosphate synthase), DXR (1-deoxy-D-xylulose 5-phosphate reductoisomerase), and MEP (2-C-methyl-D-erythritol 4-phosphate cytidylyltransferase). Downstream genes like HDR (Hydroxymethylbutenyl diphosphate reductase) also exhibited increased expression, suggesting an enhanced production of precursor compounds for terpene biosynthesis. Similarly, the MVA pathway in ‘B1’ showed elevated expression of HMGS (3-hydroxymethylglutaryl-CoA synthase), HMGR (3-hydroxy-3-methylglutaryl-CoA reductase), and TPS (Terpene Synthase) genes, indicating the active synthesis of monoterpenes and triterpenoids that are critical for pathogen resistance. In contrast, the susceptible variety ‘D30’ displayed inconsistent or downregulated expression patterns across both pathways, suggesting an impaired capacity to activate terpenoid-mediated defenses. These findings highlight the importance of terpenoid biosynthesis in enhancing disease resistance in the ‘B1’ variety.

### 2.8. qRT-PCR Verification of RNA Sequencing Results

**Figure 7:**
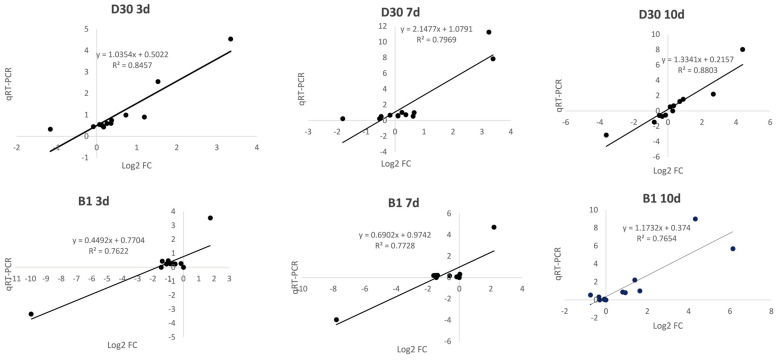
Validation of transcriptome sequencing results using qRT-PCR.

To verify the transcriptome sequencing results, twelve random DEGs with high expression levels were selected for qRT-PCR verification. The selected genes included shikimate O–hydroxycinnamoyltransferase (LOC102582263), *AUX/IAA* (LOC102587812), *bZIP* (LOC102591673), *bHLH* (LOC102599641), *MYB* (LOC102603892), pathogenesis related protein 1 (LOC102581167 and LOC102590095), *TGA* (LOC102603939), *PBS1* (LOC102585834), *RPM1* (LOC107063329), LRR receptor-like serine/threonine-protein (LOC102589771), and *R1B-14* (*LOC102583285*). The expression level of genes increased in all periods after infection by *A. solani*, and the expression of other genes showed differential changes. Although the differences in multiples of several DEGs in the results of RNA-seq and qRT-PCR were not always similar, their correlation coefficients were all above 0.75, indicating a consistent overall trend, which verified the accuracy of the results of RNA-seq (Figure 7).

## 3. Discussion

The fungus *A. solani*, causing early blight, is a devastating fungus responsible for heavy annual yield loss in potato-growing regions. Studies have shown that the overall annual yield loss can be up to 80% in some regions [38,39]. The application of recent next-generation technologies has shown that comparative omics are promising tools to identify the candidate genes and pathways associated with disease resistance in many crops [40,41,42,43]. Primary screening of plant varieties in our earlier research showed that some varieties possess high levels of resistance to early blight. We found two varieties with different levels of resistance, and virus-free seedlings were used for experimental purposes. The primary disease symptoms induced by *A. solani* infection, such as necrotic spots, yellowing of leaves, curling of leaves, and tissue damage at 3, 7, and 10 dpi, were observed between the two varieties. Severe damage in sensitive (D30) seedlings was observed after 10 dpi. Resistance was observed in the B1 variety, and mild symptoms were observed in the resistant variety. A foliage bioassay on leaflets of whole plants of the wild type (cv. Désirée) was performed, and it was found that the infection symptoms caused by the early blight fungus were similar to our study [44]. In a different study, screening of several Iranian potato cultivars demonstrated that variation in resistance exists between some cultivars. Their findings revealed that the ‘Diamond’ cultivar was more resistant to multiple isolates of *A. solani* when compared to cv. Granula [45].

According to the different number of DEGs, the specific differentially expressed genes of ‘B1’ showed a decreasing trend with the extension of infection time, and ‘B1’ exhibited strong resistance to *A. solani* infection. Meanwhile, D30 showed an increasing trend with the extension of infection time and exhibited no resistance to the infection of *A. solani*. The total number of ‘D30’-induced DEGs was higher in susceptible plants at the three infection time points (3d, 7d, and 10d), indicating strong transcriptional regulation of ‘D30’ interaction in susceptible potatoes. The diverse expression of transcripts in varieties is clearly explained by many transcriptome studies [46,47,48,49]. In another study, a comprehensive transcriptome analysis of multiple potato cultivars was performed to gain detailed insight into early blight infection. The results of their findings revealed that upregulated DEGs were twice in number compared to the downregulated ones in all the potato cultivars [33]. A similar pattern of upregulated DEGs was identified in our study, where these genes were higher in number at the later stages of disease infection. Furthermore, the GO and KEGG [37] analyses of DEGs showed that ‘B1’s resistance to *A. solani* is probably driven by a robust immune response. The susceptibility in ‘D30’ is more likely due to its failure to activate sufficient defense pathways at the early stages of infection. The substantial downregulation of key metabolic processes, which compromises their recovery and survival, was also observed. The strong regulation of GO terms and KEGG pathways related to defense, stress response, and specialized metabolites at 7 and 10 dpi was observed in the resistant variety. The comparative transcriptomics in many crops, including *Citrullus lanatus* [40], *Prunus avium* [50], and *Panax ginseng* [51], also revealed that the pathways related to defense mechanisms are activated at the early stage of infection in resistant plants. These findings strengthen our knowledge of important pathways associated with disease resistance in potatoes.

In ‘B1’ seedlings, a moderate downregulation of key processes such as photosynthesis and energy metabolism enables the plant to maintain a balance between growth and defense. In contrast, in ‘D30’ seedlings, defense-related pathways and associated Gene Ontology (GO) terms showed little to no activation following inoculation, particularly at later time points. Similarly, another study found that a susceptible response in potato varieties after exposure to root-knot nematodes was linked to a delayed induction of pathogenesis-related (PR) genes, activation of the host’s antioxidant system, and suppression of disease resistance genes [52].

The severe damage in these sensitive plants can be correlated with significant downregulation of photosynthesis and metabolic pathways post-inoculation. In addition, pathways related to photosynthesis, carbon fixation, and energy metabolism are notably downregulated in ‘D30’, particularly at early stages (3 dpi). Alternatively, in B1, these pathways are less prominently affected, suggesting that the resistant variety retains better energy production and photosynthetic activity, which is also visible in the phenotypic characterization of both varieties. In short, the ‘B1’-resistant variety demonstrates a strong activation of defense-related genes, particularly those involved in basic defense pathways like phenylpropanoid, hormone signaling, and photosynthesis recovery. On the contrary, the ‘D30’-sensitive line shows a weaker or absent induction of these processes, highlighting its sensitivity. The findings suggest that the resistance in ‘B1’ is mediated by early activation of defense pathways, structural reinforcement, and long-term recovery mechanisms, while the sensitive ‘D30’ lacks these robust responses.

WGCNA is a comprehensive analysis method that enables researchers to understand the relationships among all genes in RNA-seq, rather than examining each gene [53,54]. Additionally, WGCNA provided insights into the associations between modules and phenotypic traits in studies [55,56,57]. We performed WGCNA and found intriguing results. Multiple modules were identified in our analysis. The gene co-expression network analysis of the comparative transcriptome was performed in cultivated peanuts [58]. In line with our experimental analysis, Cui et al. (2022) identified hub genes positively associated with resistance to *A. flavus* in two genotypes using comparative transcriptome and WGCNA methods [58].

Two specific modules (lightsteelblue1 and lightpink4) were selected out of multiple modules identified. The module (lightsteelblue1) was associated with disease resistance in the ‘B1’ and contains TFs like *WRKY33* and *MYB* as key regulators. *WRKY33* is currently one of the most studied members of the Group I WRKY transcription factor family of *A. thaliana* Chen and Zhang [59] Recent studies have confirmed that *WRKY33* and its orthologous genes in other crops regulate various biological and abiotic stresses and occupy a central position in the regulatory network. For instance, *AtWRKY33* in Arabidopsis, *BnWRKY33* in *Brassica napus*, *VvWRKY33* in grapevine [60], *GhWRKY33* in *Gossypium hirsutum* [61], and *BcWRKY33A* in *Brassica campestris* [62] were found to be associated with various stress resistance in crops. The hub module (lightpink4) was associated with disease susceptibility in the ‘D30’ variety. TFs like AP2 and ERF were identified in this module. The above findings suggest that specific TFs drive regulatory networks contributing to resistance or susceptibility in potato varieties. Furthermore, the DEGs specific to various pathways were analyzed. The results revealed that ‘B1’ exhibited stronger activation of key genes like *PALs* (*LOC102587888*, *LOC107057661*, *LOC102585026*, *LOC102596343*, *LOC102582618*, *LOC102585357*, and *LOC102597781*), *4CLs* (*LOC102594991*, *LOC102581073*, and *LOC102589212*), *DFR* (*LOC102587107*), and *ANS* (*LOC102598323*), leading to enhanced flavonoid compound accumulation. The findings of Yadav et al. [40] in their comparative analysis of resistant and susceptible plants also demonstrated that genes in phenylpropanoid pathways are associated with disease resistance in plants. The genes PALs [63], 4CL [64,65,66], DFR [67,68], and ANS [69] were associated with disease resistance in plants. Few genes (*DXS*, *DXR*, HMGS, and *TPS*) from other pathways, including MEP and MVA in B1, showed higher expression. The role of the MVA pathway in the defense of potatoes after infection with early blight was also highlighted in studies by [33]. These findings suggest that the resistant variety (B1) activates both flavonoid and terpenoid biosynthesis pathways more effectively, contributing to its robust defense mechanism against *A. solani* infection. By identifying resistance-associated genes and key signaling pathways involved in defense against *Alternaria solani*, our findings offer molecular targets for breeding programs aiming to develop potato cultivars with improved resistance to early blight. Furthermore, the differentially expressed genes identified, such as *LOC102603761* and *LOC102573998*, may serve as useful molecular markers for early screening of resistant varieties, thereby enhancing the efficiency of selection. Beyond breeding, insights into the molecular mechanisms, particularly the role of flavonoid biosynthesis and transcription factors like WRKY33 and MYB, can inform the development of novel, targeted strategies for integrated disease management. Ultimately, the research contributes to more sustainable potato production by supporting efforts to reduce yield losses and reliance on chemical fungicides through the use of resistant cultivars.

In short, it was identified that genes from multiple pathways are responsible for disease resistance in potatoes. Our findings provide robust evidence supporting the use of transcriptomic methods for gene mining, as well as identifying candidate genes and various pathways that hold significant potential for further studies aimed at breeding purposes in potatoes. Building on our current results, future studies should focus on the functional characterization of candidate genes and transcription factors, such as WRKY33, MYB, NAC, and other key hub genes identified through WGCNA, employing approaches like gene knockout or overexpression to confirm their specific roles in resistance. We also propose conducting multi-environmental field trials to evaluate the effectiveness and durability of resistance traits under variable conditions. Furthermore, investigating whether these resistance mechanisms confer cross-protection against other related pathogens could broaden the impact of our findings. Finally, parallel studies on the population genetics of *A. solani* could help monitor pathogen evolution and ensure the long-term efficacy of resistance genes deployed in breeding programs.

## 4. Materials and Methods

### 4.1. Plant Varieties, Test Strains, and Inoculation Method

Virus-free seedlings of the early blight-resistant variety ‘B1’ and the susceptible variety ‘D30’ were sourced from the Crop Virus-Free and Rapid Propagation Center, Comprehensive Test Field, Xinjiang Academy of Agricultural Sciences, Urumqi, China. *A. solani* pathogen, the pathogen responsible for early blight, was also obtained from the same center. The collected and purified strains were transferred to the PDA plate medium under sterile conditions in the laboratory. They were stored at room temperature and cultured under light for 14 days to generate conidia. Sterile water was injected into the plate and filtered under sterile conditions [70]. The conidia suspension was collected and diluted [71]. At the point when the disease-resistant variety ‘B1’ and the susceptible variety ‘D30’ grew to 7~8 leaves, 10 μL was used with a pipette in a sterile environment and inoculated into the leaves without touching the bottom of the virus-free seedlings. Ten plants were inoculated in each variety for each time point, and sterile water was used as the control. The same procedure was repeated three times, and the temperature was maintained at 25 °C.

### 4.2. RNA Extraction

Leaf samples were collected in triplicate. The total RNA extraction was performed following the instructions provided on the RNAprep Pure Plant Plus Kit (TIANGEN, DP441, Beijing, China) [72]. RNA integrity, purity, and concentration for each sample were assessed using the Qsep400 bioanalyzer (BiOptic Inc., New Taipei City, Taiwan), the NanoPhotometer spectrophotometer (Scimetrics, Inc., Suite E200, Katy, TX, USA), and agarose gel electrophoresis [73]. The original data generated in the present study can be downloaded from the BioProject accession PRJNA1207131.

### 4.3. Library Construction and High-Throughput Sequencing

The first cDNA strand was synthesized using 6-base random hexamers based on the fragment RNA template. Subsequently, the buffer, dNTPs (dTTP, dATP, dGTP, and dCTP), and DNA polymerase-I were added to synthesize the double-stranded cDNA, and the double-stranded cDNA was purified using DNA purification magnetic beads. The purified double-stranded cDNA was then end-repaired [74]. The fragment size was selected using DNA purification magnetic beads, and the final cDNA library was obtained via PCR enrichment. After the library was constructed, the quality of the library was tested by the following methods: (1) Preliminary quantification was performed using the Qubit dye method, and the insert size of the library was detected using a fragment analyzer. The next experiment could be carried out only after the insert size met the expectation; (2) Q-PCR method was used to accurately quantify the effective concentration of the library (the effective concentration of the library was >2nM), and the library inspection was completed. After the library check was qualified, different libraries were pooled according to the target on-machine data amount and sequenced on the Illumina platform.

### 4.4. Data Filtering and Gene Annotation

Clean Reads were obtained by filtering the original data using standard procedures. Clean Reads were compared with the reference genome SolTub_3.0 (GCF_000226075.1_SolTub_3.0_genomic.fna.gz) [75] using HISAT2 [76].

### 4.5. Differential Gene Analysis and GSEA Enrichment Analysis

Analysis of expression is usually standardized by FPKM value. In reference transcriptomes, genes with FPKM values above different thresholds (≥100, ≥10, ≥1, etc.) are generally considered to be expressed. DESeq was usually used for differential analysis of gene expressions, and the conditions for screening differentially expressed genes were as follows: expression difference multiples |Log_2_ (FoldChange)| ≥ 1, FDR < 0.05 [77]. Gene Set Enrichment Analysis (GSEA) was performed using the clusterProfiler package in R to compare the control group and the treatment group at different periods. The gene list was ranked based on the metric derived from DESeq analysis (typically by log2 fold change). The enrichment results related to the phenotype of the treatment group were screened using the following significance thresholds: nominal *p*-value (*p*-value) ≤ 0.05 and false discovery rate (FDR, P-adjust) ≤ 0.25 (default parameters for significance in GSEA) [78]. Gene sets with |Normalized Enrichment Score (NES)| > 1 were considered enriched. Results were visualized using functions within the enrich plot packages.

### 4.6. Enrichment Analysis of GO and KEGG Pathways

The enrichment analysis of GO (Gene Ontology) [79] and KEGG (https://www.genome.jp/kegg/) (accessed on 15 July 2024) [37] pathway of differentially expressed genes was conducted using the cluster Profiler R package (4.10.1). A *p*-value less than or equal to 0.05 was significantly enriched.

### 4.7. Weighted Gene Co-Expression Network Analysis (WGCNA) and Prediction of Hub Gene Network

Scale-free topology network was constructed using a 12 soft threshold power (β) [57]. Further, 13,636 genes were used for WGCNA with log2 (FPKM+1) as the input to generate a network co-expression between transcription factors (TF) and non-TFs. TFs can regulate target genes by binding to specific transcription factor binding sites (TFBSs), which are observed to be highly conservative in plants. The modules were selected, and network modules were visualized using Gephi software (v0.9.2) [80].

### 4.8. Real-Time Fluorescence Quantification

To verify the results of RNA-seq, real-time fluorescence quantitative qRT-PCR was performed with the same sample. Using cDNA as template and Stef1α as internal reference gene, gene expression data were standardized, and reverse transcriptional quantitative PCR analysis of specific gene expression was performed in LightCycler (F. Hoffmann-La Roche Ltd., Basel, Switzerland) according to the manufacturer’s operating guidelines [81]. Primer 6.0 software was used to design primers, and a blast comparison was performed to confirm the specificity of primer sequences [82]. The primers were synthesized by MetWare Metabolic Biotechnology Co., Ltd., Wuhan, Hubei, China. The relative expression level of the target genes was calculated using the formula 2^−ΔΔCT^, with values representing the average of three biological repeats [83]. A total of 12 genes were screened for the validation of RNA-seq results. The primer sequence is shown in Table S1.

### 4.9. Data Statistics

GraphPad Prism version 8.4 was used to perform statistical tests, and graphs of qRT-PCR were generated. The data were analyzed using IBM SPSS Statistics (Version 12.5), and the results were subjected to a multiple mean comparison and significance (IBM, 2004). The graphs and plots related to RNA-seq are generated in the current study using R.

## 5. Conclusions

In this study, a comprehensive transcriptome analysis was carried out on potato seedlings infected with *A. solani* with different resistance levels. The ‘B1’ variety revealed its defense mechanism at 3 days post-infection, deepening the understanding of key genes and pathways in ‘B1’ and ‘D30’ potato varieties. By comparing the expression levels of ‘B1’ and ‘D30’, we revealed that the resistance mechanism of ‘B1’ was against *A. solani* infection. Significant transcriptional differences, including complex cellular, biochemical, and molecular defense responses, were identified by comparing ‘B1’ and ‘D30’ with the control, respectively. ‘B1’ showed strong defense, mainly due to the upregulated expression of genes related to both phenylpropanoid/flavonoid and terpenoid biosynthesis pathways. In contrast, the disease-susceptible variety ‘D30’ exhibits delayed or lower gene activation across these pathways, leading to weaker metabolic defenses. These results provide valuable insights into the molecular mechanisms underlying resistance and susceptibility to *A. solani* infection in potato varieties.

## Data Availability

The data generated for the current study were submitted to the public domain. Current study data will be available from NCBI under the BioProject accession PRJNA1207131. Data supporting the findings of this work are available within the paper and its Supplementary Information files.

## Supplementary Materials

The following supporting information can be downloaded at: https://www.mdpi.com/article/10.3390/plants14152422/s1, Table S1: Primer sequences of the genes selected for qRT-PCR; Table S2: Details of the transcriptomic samples; Table S3: Details of the gene mapped on database; Table S4: DEGs in specific pathways.
